# Probable first report of a motor deafferentation syndrome in the Paraguayan War

**DOI:** 10.1590/0004-282X-ANP-2020-0479

**Published:** 2021-06-14

**Authors:** Marleide da Mota GOMES, Marcos Raimundo Gomes de FREITAS

**Affiliations:** 1 Universidade Federal do Rio de Janeiro, Instituto de Neurologia, Rio de Janeiro RJ, Brazil. Universidade Federal do Rio de Janeiro Universidade Federal do Rio de Janeiro Instituto de Neurologia Rio de Janeiro RJ Brazil; 2 Universidade Federal do Rio de Janeiro, Instituto de Psiquiatria, Laboratório de História da Psiquiatria, Neurologia e Saúde Mental, Rio de Janeiro RJ, Brazil. Universidade Federal do Rio de Janeiro Universidade Federal do Rio de Janeiro Instituto de Psiquiatria Laboratório de História da Psiquiatria, Neurologia e Saúde Mental Rio de Janeiro RJ Brazil; 3 Universidade Federal Fluminense, Rio de Janeiro RJ, Brazil. Universidade Federal Fluminense Universidade Federal Fluminense Rio de Janeiro RJ Brazil

**Keywords:** Beriberi, Polyneuropathy, Brain Diseases, Myelinolysis, Central Pontine, Thiamine Deficiency, Locked-in Syndrome, Beriberi, Polineuropatias, Encefalopatias, Mielinólise Central da Ponte, Deficiência de Tiamina, Síndrome do Encarceramento

## Abstract

The Paraguayan War ended 150 years ago. Back then, there were outbreaks of combatants’ limb weakness and tingling related to "palustrian cachexia", not clearly funded at the time on nutritional deficiency, the use of native flora to feed troops, and alcoholism. We report a case of a soldier with ascending paralysis, mental confusion and finally tetraplegia with preserved oculomotricity. This would probably be a case of locked-in syndrome (LIS) due to Gayet-Wernicke's encephalopathy consequent to thiamine deficiency. The role of thiamine in the peripheral or central nervous system expression was shown decades later to be related to poor diet, or use of foods containing thiaminase or thiamine antagonists, worsened by the fact that the bodily stores of thiamine are restricted, and deficits may grow fast.

## RESUMO

A Guerra do Paraguai terminou há 150 anos. Houve surtos de fraqueza dos membros dos combatentes e parestesias relacionadas à “caquexia palustre”, não claramente fundamentados na época sobre deficiência nutricional, o uso da flora nativa para alimentar as tropas e o alcoolismo. Referimo-nos a um caso de um militar com paralisia ascendente, confusão mental e finalmente tetraplegia com oculomotricidade preservada. Esse relato seria provavelmente devido a um caso de “síndrome do encarceramento” secundário à encefalopatia de Gayet-Wernicke consequente à deficiência de tiamina. O papel da tiamina na expressão periférica ou do SNC foi previsto décadas mais tarde em relação a uma dieta pobre, ou ao uso de alimentos contendo tiamina ou antagonistas da tiamina, agravado pelo fato de que as reservas corporais de tiamina são restritas, e os déficits podem se manifestar rapidamente.

**Figure 1.**
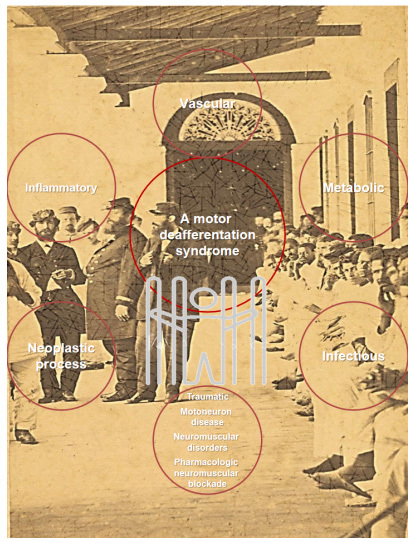
Motor deafferentation syndrome etiologies^9^, including the central pontine-myelinolysis and the Gayet-Wernicke encephalopathy caused by thiamine deficiency reported in the Paraguayan War as a hypothesis, and, in the backgroung, original photo at the church Tuyu Cué (Neembucu, Paraguai) serving in the Brazilian Infirmary.

Source: digital collection of Fundação Biblioteca Nacional.

The locked-in syndrome (LIS), also known as motor deafferentation syndrome, is a rare neurological disorder that was first described by Plum and Posner^1^ in 1966, and which is characterized by quadriplegia and anarthria with preserved consciousness. The syndrome was considered to have many etiologies (Figure 1).

**Figure 2.**
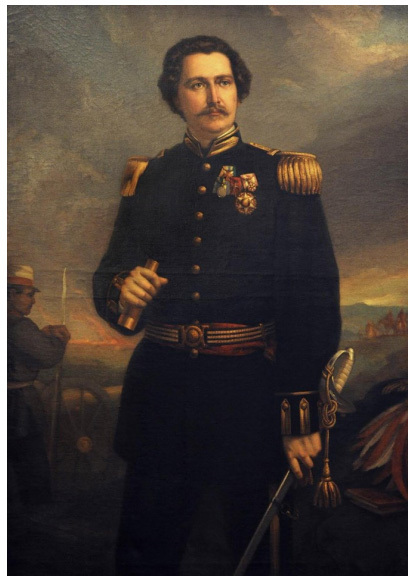
Alfredo d’Escragnolle Taunay, Visconde de Taunay (1843-1899).

Source: Reproduction of original picture by Louis Auguste Moreau (public domain).

We emphasize some aspects of a historical vignette that looks like a LIS description. This is a very peculiar clinical picture described by an academic aristocrat of the Brazilian Army, the author of Memorias^2^: Alfredo Maria Adriano d’Escragnolle Taunay, visconde de Taunay (Figure 2). Taunay was also a writer, musician, teacher, military engineer, politician, historian and Brazilian sociologist. He reports the case of a Brazilian soldier who presented an ascending paralysis, mental confusion, followed by an apparent lucidity associated with quadriplegia (Box 1).

**Box 1.** Case reported by Alfredo d’Escragnolle Taunay, published posthumously^2^, and probably one of the first cases of Locked-in syndrome (LIS) in the literature

“You cannot imagine what I am suffering. It is a pain of agony, nor is there any other that is comparable to it. Death is rising! See how cold and immobile feet and legs are”. And, in fact, as he spoke, their limbs were stiffened. “Now, it's the arms!” And he stood with them straight, as if they were made of stone. […] If you shut up, it was for a short time; the moans and cries started again [...] At last, he stopped, but when paralysis caught his tongue and lips. And he was stretched out, stiff and immobile, on the death cot […] like a marble statue of those who sleep in the tombs of the Middle Ages. Only his eyes swam in his orbits, still indicating life and horrible anxieties, as tears flowed from them, which wet the pillow. The unfortunate remained for a day and a half until he exhaled his last breath at one o’clock on Jul 26, 1866”.

Before discussing this case, we must remember that the Paraguayan War ended 150 years ago, and it occurred mostly in the extreme southwest of Brazil and in Paraguay. Consequently, military logistics deficiency led the troops to severe food deprivation and related scenarios, as the one here presented.

At this wartime, outbreaks of combatants’ limb weakness with particular features such as tingling occurred. This was supposedly due to “palustrian causes”. However, there was a shortage of supply, poor environmental conditions, and diarrheal diseases, besides reports of native flora being used to feed troops and alcoholism. There were also accounts on the death of horses with symptoms similar to that of combatants^2^^,^^3^^,^^4^.

At that time, Science did not have all the critical clinical elements to establish the diagnosis of thiamine deficiency due to inadequate intake, food with anti-thiamine factors, or alcoholism, besides rare genetic cases^5^. Regardless of the underlying cause, thiamine deficits may have severe detrimental effects, with most of the symptoms manifesting at the neurological level^5^. However, far from the war front, Silva Lima was studying beriberi^6^. He had already identified, then, similarities between cases he assisted and the war cases^7^.

We scrutinized the Brazilian troop neuropathic outbreaks considering several scenarios in this study, but mainly this peculiar case. This would be one of the first reported in the literature that sheds light on an underestimated part of the history of Neurology in wartime.

This soldier, apparently with delirious and rapidly evolving tetraparesis, but with the maintenance of eye movements, may have had a LIS. This syndrome is linked to several etiologies; it can stem from basilar artery occlusion by stroke, Gayet-Wernicke encephalopathy (GWE) with central pontine myelinolysis (CPM) due to thiamine deficiency, a form of dry beriberi, and the Guillain-Barré syndrome (GBS).

About 82% of patients with GWE present with delirium, as reported by Osiezagha et al.^8^ based on a case series of autopsies. As for the LIS, it is expressed by sustained eyelid opening, preserved necessary cognitive abilities, severe aphonia or hypophonia, quadriplegia or quadriparesis, and a primary mode of communication that uses vertical or lateral eye movement, or upper eyelid blinking^9^.

Regarding the progressive ascending motor paralysis of the reported case, the GBS should also be considered. This is an immune-mediated disease of peripheral nerves and nerve roots that is often activated by infections, which is very common in wartime^10^. The progressive phase of GBS usually lasts from two days to four weeks. Consequently, in patients who reach maximum disability within 24 hours after the onset of the disease, as supposedly occurred in the reported case, alternative diagnoses should be contemplated. Likewise, diagnoses related to altered consciousness may be considered - except Bickerstaff's brain stem encephalitis, a variant of GS with the involvement of cranial nerves.

Besides, a peripheral disconnection syndrome, which can occur with GBS and severe post-infection polyneuropathy, would include an external ophthalmoplegia, apparently not present in the reported case^9^.

In contrast, dry beriberi may mimic the most common form of GBS, and polyneuropathy secondary to thiamine deficiency may develop gradually over weeks to months, but also acutely, and consequently may be confused with GBS^10^. This polyneuritis can also be associated with GWE by thiamine deficiency, as presented by Shible et al.^11^.

Regarding the reported manifestations of irritability, restlessness, and complaint of intense suffering, with a note about stiffness (muscle spasms?), generalized tetanus should also be examined^12^. Once again, a comment on the report “[...] After all, the doctor reminded him to give him calomel”. In fact, mercury compounds, like calomel, were used in medicinal preparations in the past. However, this toxic cause is less likely for this discussed case: “Toxic effects were soon noticed in individuals given large doses for long periods [...] They had troubling neurologic symptoms, such as arm and facial tremors, hyporeflexia, weakness, ataxia, and erethism [...]”^13^.

In any case, considering the immobility emphasized by the writer, and the significant probability of diagnosis related to the high prevalence of thiamine deficiency, the most likely diagnosis for the reported case would be GWE with CPM, less likely GBS. Coincidently, GBS was first described in the World War I by Georges Guillain, Jean Alexandre Barré, and André Strohl when they witnessed (1916) two similar cases of soldiers who had partial paralysis with significant impairment of reflexes with spontaneous regression^14^. However, both GWE with CPM and GBS have some characteristics of motor deafferentation syndrome or LIS that may encompass many etiologies (Figure 2).

This case report is similar to that of Alexandre Dumas in “The Count of Monte Cristo” (1844), who was “a corpse with living eyes”, and one by Emile Zola, in his novel “Thérèse Raquin” (1868)^9^. However, the first case of unmoving physical body, a deafferented patient, in medical literature was described by Darolles (1875)^9^. Consequently, the case witnessed by Taunay in 1866 and only later on published would be one of the first to recognize the LIS in its initial phase.

This detailed historical research can be useful, as one can learn from the past. The description by Taunay leads us to believe we are addressing LIS or motor deafferentation syndrome due to thiamine deficiency. It is more likely to happen in adverse conditions such as war and in the early phase of LIS, since its diagnosis is usually only noticed later by attentive caregivers.
